# *In situ *origin of deep rooting lineages of mitochondrial Macrohaplogroup 'M' in India

**DOI:** 10.1186/1471-2164-7-151

**Published:** 2006-06-15

**Authors:** Kumarasamy Thangaraj, Gyaneshwer Chaubey, Vijay Kumar Singh, Ayyasamy Vanniarajan, Ismail Thanseem, Alla G Reddy, Lalji Singh

**Affiliations:** 1 Centre for Cellular and Molecular Biology, Hyderabad-500 007, India; 2 Department of Evolutionary Biology, Institute of Molecular and Cell Biology, University of Tartu and Estonian Biocentre, Tartu, Estonia

## Abstract

**Background:**

Macrohaplogroups 'M' and 'N' have evolved almost in parallel from a founder haplogroup L3. Macrohaplogroup N in India has already been defined in previous studies and recently the macrohaplogroup M among the Indian populations has been characterized. In this study, we attempted to reconstruct and re-evaluate the phylogeny of Macrohaplogroup M, which harbors more than 60% of the Indian mtDNA lineage, and to shed light on the origin of its deep rooting haplogroups.

**Results:**

Using 11 whole mtDNA and 2231 partial coding sequence of Indian M lineage selected from 8670 HVS1 sequences across India, we have reconstructed the tree including Andamanese-specific lineage M31 and calculated the time depth of all the nodes. We defined one novel haplogroup M41, and revised the classification of haplogroups M3, M18, and M31.

**Conclusion:**

Our result indicates that the Indian mtDNA pool consists of several deep rooting lineages of macrohaplogroup 'M' suggesting *in-situ *origin of these haplogroups in South Asia, most likely in the India. These deep rooting lineages are not language specific and spread over all the language groups in India. Moreover, our reanalysis of the Andamanese-specific lineage M31 suggests population specific two clear-cut subclades (M31a1 and M31a2). Onge and Jarwa share M31a1 branch while M31a2 clade is present in only Great Andamanese individuals. Overall our study supported the one wave, rapid dispersal theory of modern humans along the Asian coast.

## Background

Variability in human mitochondrial DNA (mtDNA) offers valuable information to trace the genetic history of humans, because of the high rate of mutation and absence of recombination. Analyses of the frequency of variation and distribution of mtDNA haplogroups have been used to evaluate current models concerning the process of colonization of world. South Asia, particularly India, lies on the path of the earliest human dispersal from Africa [1,2] and hence this holds an important site of information on early human migrations.

Macrohaplogroups 'M' and 'N' have evolved almost in parallel from a founder haplogroup L3. A number of mtDNA studies based on HVS I in Indian populations have been carried out, and some information have been made available about the genetic structure of Indian gene pool [3-9]. Macrohaplogroup N in India has already been defined [10], and recently the macrohaplogroup M among the Indian populations has also been characterized [11].

The origin of mtDNA macrohaplogroup M has been an issue of controversy. Macrohaplogroup M is found mainly in Asia, and its various clades makeup the great majority of Indian and Mongoloid lineages. It was hypothesized that its high frequency and diversity in Ethiopia may indicate an African origin for the entire M [14]. Nevertheless, M is geographically limited to Africa, while it is prevalent in Asia. If M originated in Africa, then it must have occurred at an ancient time, since it had to spread throughout Asia and the New World. However, it is paradoxical that M crossed such a vast distance and failed to accomplish other populations of Africa, except Ethiopians and few Egyptians. The lack of time depth and classification of subhalogroups in the case of most of the lineages in the recent paper [11] tends us to reconstruct the phylogeny of macrohaplogroup M, which harbors more than 60% of the Indian mtDNA lineage [1,3-9].

## Results and discussion

**Figure 1:**
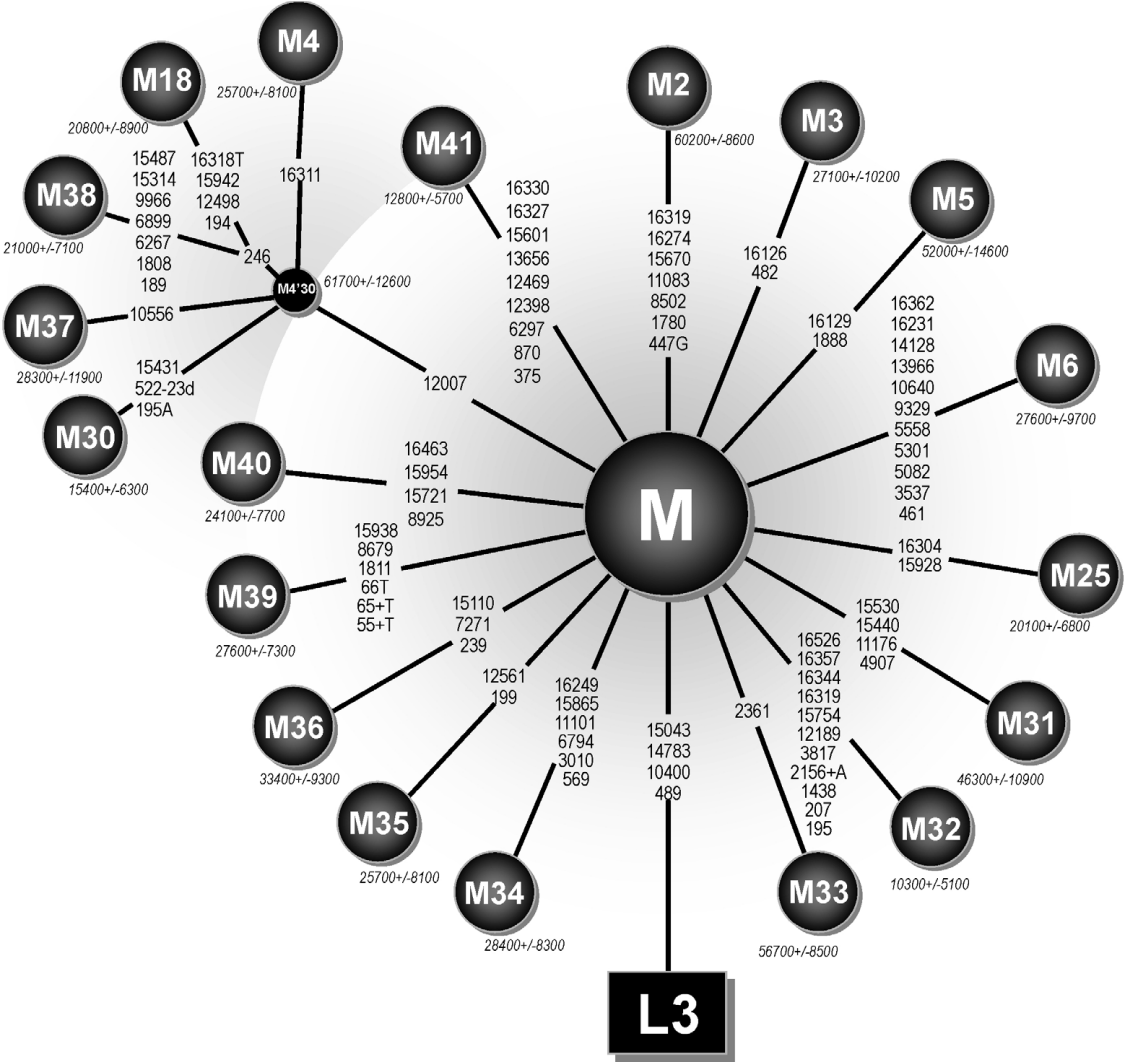
The 'autochthonous' haplogroups of Indian macrohaplogroup M.

**Figure 2:**
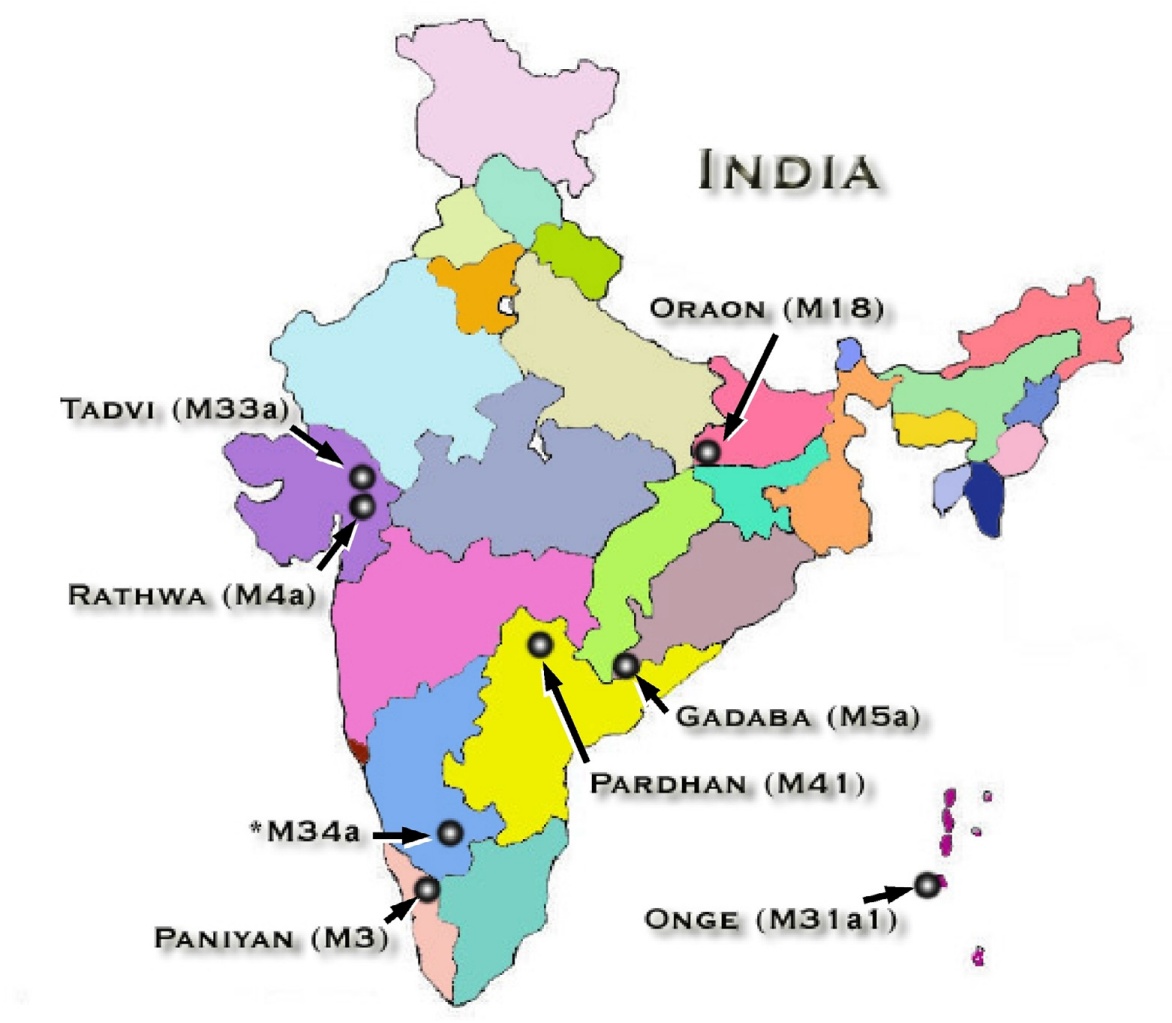
Geographical distribution of completely sequenced (mtDNA) samples used for the reconstruction of Indian M phylogeny. (*) Random sample from Karnataka state.

In this study we defined one novel haplogroup M41, and revised the classification of haplogroups M3, M18, and M31. The remaining haplogroups are also classified into subhaplogroups (M3a, M4a, M6b, M33a, M34a, M37a and M40a) by including our complete sequence information (Fig. 1 and also see Additional file 1). The geographical distributions of these samples with their haplogroup affiliation are given in Fig. 2 and the populations and their linguistic affiliations are described in Table 1.

We have revised the classification of haplogroup M3 that was previously characterized by a coding region mutation 4580 and two control region substitutions 482 and 16126. In our survey of >5000 samples across India, we found a considerable number of samples that have mutations at nps 482 and 16126 but don't have mutation at 4580 (our unpublished data). This suggests that 482 and 16126 are the basal mutations of this haplogroup, and 4580 might have originated later and this represent subhaplogroup M3a. Haplogroup, M18 was previously characterized by only the HVS I mutation (16318T), but now we have defined this haplogroup by two coding region mutations (12498 and 15942), and an additional control region mutation (194) (Fig. 1 and also see Additional file 1).

Further, we have defined several subhaplogroups based on the sharing mutations between our own and Sun et al. [11] data [see Additional file 1]. Subhaplogroup M3a defined by a coding region mutation at 4580, M4a defined by two coding (6620 and 7859) and three control region (152, 16145 and 16261) mutations. Subhaplogroup M6b defined by two coding region mutations (3486 and 5585). M33a defined by two coding substitution (8562 and 15908); and M34a by six coding region (3447-8404-10361-11992-12311-14094) and three control region (146, 16095 and 16359) mutations. M37a defined by a single coding (7853) and two control region (151-152) mutations. M40a defined by a coding (13542) and three control region (200, 16179 and 16294) mutations. Interestingly, haplogroups M4, M18, M30, M37 and M38 shared a common coding region mutation (12007) from the root of haplogroup M (superhaplogroup M4'30) and later differentiated by coding and control region mutations (Fig. 1 and also see Additional file 1). We have defined one novel haplogroup, designated as M41, by six coding (870-6297-12398-12469-13656-15601) and three control (375, 16327 and 16330) region mutations with the T159 sequence of Sun et al. [11] (Fig. 1 and also see Additional file 1). M41 has also been tentatively classified in three subhaplogroups M41a, M41b and M41c. One complete sequence O9 from Andamanese specific haplogroup M31a has also been included here that defines subhaplogroup M31a1 by a single coding (13710) as well as a control region (200) substitution. Our reanalysis of this lineage suggests population-specific two clear-cut subclades of M31a in this island. M31a1 specific mutations 200 and 13710 are exclusively present in Onge and Jarwa populations while 9617 defining M31a2 clade is present in only Greater Andamanese individuals [see Additional file 1]. All the coding region diagnostic mutations of macrohaplogroup M subhaplogroups are listed in Table 2.

We have also calculated the age estimates for all M branches (Fig. 1) both by using the estimated mutation calibration rate of Mishmar et al. [16], which has been recently applied in most of the mtDNA studies [1,2,17], and ρ (the averaged distance to a specified founder haplotype) and a mutation rate of one transition per 20,180 years between nps 16090–16365 [18]. Standard errors for coalescence time calculation were calculated following Saillard et al [19]. We found some conflict between the age estimated by both of the methods. Since, the complete sequences do not reflect the actual population size and geographical distribution, former method [18] has been used for colescent time estimation. The detailed coalescent time list is given in Additional file 2.

It is interesting to note that most of the new M lineages are deep rooting, and more likely arose *in situ *in the Indian subcontinent just after the arrival of the anatomically modern humans (Fig. 1). As shown in the figure 1, it is apparent that all the autochthonous lineages under analysis emerge directly from the root of the macrohaplogroup M. There is no intermediate lineage shared by any two haplogroups, except for haplogroup M4'30 (Fig. 1). The star-like and non-overlapping pattern (Fig. 1) indicates that all the lineages have originated independently from the root of the macrohaplogroup M, thus supporting a rapid dispersal of modern humans along the Asian coast after they left Africa, followed by a long period of isolation [2].

## Conclusion

In summary, based on well-resolved mtDNA macrohaplogroup M phylogeny, it can be confirmed with the recent studies [1,2] that a rapid dispersal of modern human took place in one wave along the Asian coast. The deep roots of M phylogeny clearly ascertain the relic of Indian lineages as compared to other M sub lineages suggesting '*in-situ*' origin of these sub-haplogroups in South Asia, most likely in India. These deep rooting lineages are not language specific and spread over all the language groups in India. Moreover, our reanalysis of Andamanese-specific lineage M31 suggests population specific two clear-cut subclades. Onge and Jarwa share M31a1 branch while M31a2 clade is present only in Great Andamanese individuals [see Additional file 1].

## Methods

A phylogenetic tree reconstructed from the data of Sun et al [11] including our complete sequence information of 11 complete sequences and 2231 partial Indian samples possessing M selected from 8670 HVS sequences across India to resolve some of the anomalies arising due to recurrent mutations in the control region. The geographical distributions of the samples that are used for the complete sequencing of mtDNA are given in table 2. We followed the experimental procedures, quality control measures and haplogroup nomenclature described in our previous study [1]. We have made the parsimonious tree by using our own data and data published elsewhere [11-13,15,21,22]. The maximum parsimonious tree obtained by this procedure is shown in the figure 1 and Additional file 1.

## Electronic database information

Accession numbers [23] for data presented herein are as follows (for the complete mtDNA sequence accession numbers DQ408672-DQ408680 and DQ513521-DQ513522, and for the partial region sequence accession numbers DQ653413-DQ655643).

## Competing interests

The author(s) declare that they have no competing interests.

## Authors' contributions

LS, KT, and GC conceived and designed the experiments. GC, VK, IT, AV and AGR performed the experiments and analyzed the data. LS, KT and GC wrote the paper.

## Supplementary Material

Additional File 1 Reconstructed phylogeny of macrohaplogroup M tree by using our data and data published elsewhere [11-13,15,21,22] of Indian complete mtDNA sequences. Mutations are scored after comparing with r-CRS [20]. Notorious mutation 16519 has been excluded from the analysis. Recurrent mutations are underlined. Suffixes are transversions. The literature samples are given with the name of first author. Click here for file

Additional File 2 Estimated coalescent time of all the Indian M sublineages. Click here for file

## Figures and Tables

**Table 1:** Samples that were sequenced for the complete mitochondrial DNA and their geographical location, linguistic affiliations and haplogroups assigned,

**Sl. No.**	**Sample Code**	**Haplogroup**	**Population**	**Location**	**Linguistic Affiliation**
1	P12	M3	Paniya	Kerala	Dravidian
2	Or89	M5a	Gadaba	Orrisa	Austro-Asiatic
3	P31	M6b	Paniya	Kerala	Dravidian
4	T9	M33a	Tadvi	Gujarat	Indo-European
5	M42	M34a	*Random*	Karnataka	Dravidian
6	Or82	M18	Oraon	Bihar	Austro-Asiatic
7	T12	M4a	Tadvi	Gujarat	Indo-European
8	R1	M37a	Rathwa	Gujarat	Indo-European
9	PS38	M41b	Pardhan	Andhra-Pradesh	Dravidian
10	PJ10	M41c	Pardhan	Andhra-Pradesh	Dravidian
11	O9	M31a1	Onge	Andaman	Andamanese

**Table 2:** Coding region diagnostic mutations of Indian (mtDNA) M subhaplogroups.

**Haplogroups**	**Diagnostic coding region substitutions**
M2	447G-1780-8502-11083-15670
M2a	5252-7961-8396-9758-12810
M3	482
M3a	4580
M4a	6620-7859
M4b	511
M5	1888
M5a	709-3921-12477-14323
M6	461-3537-5082-5301-5558-9329-10640-13966-14128
M6b	5585-3486
M18	12498-15942
M25	15928
M30	15431
M30a	6366
M30b	5147-13980
M30c	12234
M30d	15259
M31	4907-11176-15440-15530
M32	3817-9064-12189-15754
M33	2361
M33a	8562-15908
M34	569-3010-6794-11101-15865
M34a	3447-8404-10361-11992-12311-14094
M35	12561
M35a	5432-10670-15924
M36	7271-15110
M37	10556
M37a	7853
M38	1808-6367-6899-9966-15314-15487
M39	1811-8679-15938
M40	8925-15721-15954
M40a	13542
M41	870-6297-12398-12469-13656-15601
