# Supplementary figures and images for: In situ origin of deep rooting lineages of mitochondrial Macrohaplogroup 'M' in India
Source: BMC Genomics. 2006 Jun 15;7:151. doi: 10.1186/1471-2164-7-151 (PMC1534032; doi:10.1186/1471-2164-7-151)

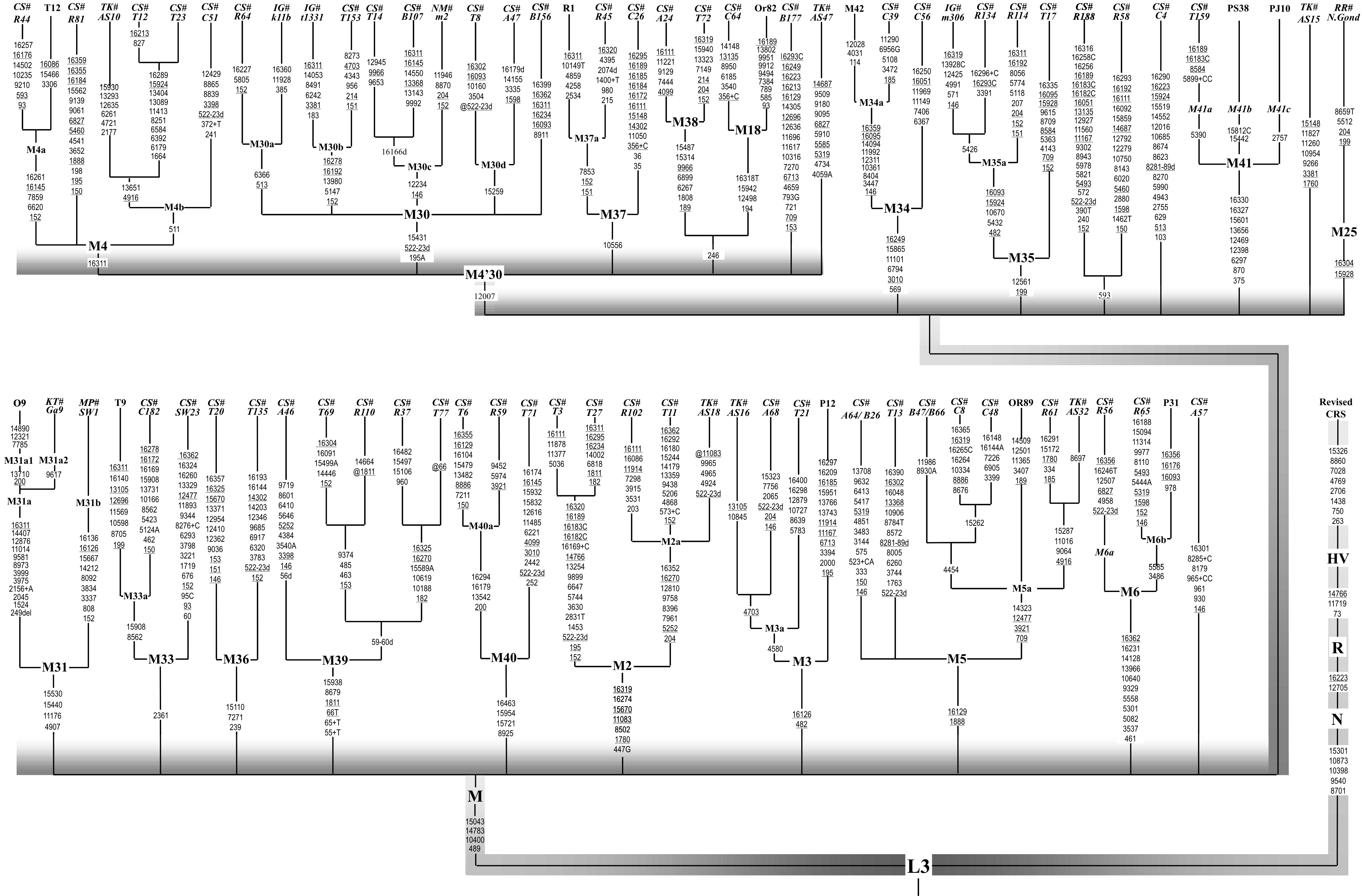

Supplement: Additional File 1 — Reconstructed phylogeny of macrohaplogroup M tree by using our data and data published elsewhere [11-13,15,21,22] of Indian complete mtDNA sequences. Mutations are scored after comparing with r-CRS [20]. Notorious mutation 16519 has been excluded from the analysis. Recurrent mutations are underlined. Suffixes are transversions. The literature samples are given with the name of first author. [file 1471-2164-7-151-S1.jpeg]
