# Supplementary material for: In situ origin of deep rooting lineages of mitochondrial Macrohaplogroup 'M' in India
Source: BMC Genomics. 2006 Jun 15;7:151. doi: 10.1186/1471-2164-7-151 (PMC1534032; doi:10.1186/1471-2164-7-151)
Supplement: Additional File 2 — Estimated coalescent time of all the Indian M sublineages. [file 1471-2164-7-151-S2.doc]

| **Haplogroup** | **Sample size** | **ρ±σ** | **Time (Years)x1000** |
| --- | --- | --- | --- |
| M2 | 76 | 3.0+0.43 | 60.2+/-8.6 |
| M2a | 24 | 1.9+0.50 | 38+/-10.2 |
| M3 | 40 | 1.3+0.50 | 27.1+/-10.2 |
| M3a | 48 | 0.8+0.30 | 16.4+/6.1 |
| M4 | 52 | 1.3+0.40 | 25.7+/-8.1 |
| M4a | 18 | 0.8+0.32 | 15.4+/-6.3 |
| M4b | 18 | 0.8+0.32 | 15.4+/-6.3 |
| M5 | 34 | 2.6+0.72 | 52+/-14.6 |
| M5a | 23 | 1.1+0.38 | 23.1+/-7.7 |
| M6 | 17 | 1.4+0.48 | 27.6+/-9.7 |
| M6a | 9 | 1.1+0.38 | 23.1+/-7.7 |
| M18 | 22 | 1.0+0.44 | 20.8+/-8.9 |
| M25 | 12 | 1.0+0.34 | 20.1+/-6.8 |
| M30 | 24 | 0.8+0.32 | 15.4+/-6.3 |
| M30a | 11 | 0.3+0.18 | 5.1+/-3.6 |
| M30b | 10 | 0.2+0.14 | 4.177+/-2.8 |
| M30c | 11 | 0.3+0.18 | 5.1+/-3.6 |
| M30d | 12 | 0.6+0.28 | 12.8+/-5.7 |
| M33 | 34 | 2.8+0.42 | 56.7+/-8.5 |
| M33a | 14 | 1.4+0.25 | 28.4+/-5.1 |
| M34 | 22 | 1.4+0.41 | 28.4+/-8.3 |
| M34a | 21 | 0.8+0.32 | 15.4+/-6.3 |
| M35 | 18 | 1.3+0.40 | 25.7+/-8.1 |
| M35a | 17 | 0.5+0.25 | 10.3+/-5.1 |
| M36 | 14 | 1.7+0.46 | 33.4+/-9.3 |
| M37 | 17 | 1.4+0.59 | 28.3+/-11.9 |
| M37a | 16 | 0.9+0.34 | 18+/-6.8 |
| M38 | 15 | 1.0+0.35 | 21+/-7.1 |
| M39 | 13 | 1.4+0.36 | 27.6+/-7.3 |
| M40 | 21 | 1.2+0.38 | 24.1+/-7.7 |
| M40a | 18 | 0.9+0.34 | 18+/-6.8 |
| M41 | 23 | 0.6+0.28 | 12.8+/-5.7 |
| M4'30 | 65 | 3.1+0.62 | 61.7+/-12.6 |
